# Prevalence and risk factors of feline lower urinary tract disease in Chiang Mai, Thailand

**DOI:** 10.1038/s41598-019-56968-w

**Published:** 2020-01-13

**Authors:** Kakanang Piyarungsri, Sahatchai Tangtrongsup, Niyada Thitaram, Phatthamaporn Lekklar, Atiratt Kittinuntasilp

**Affiliations:** 1 Department of Companion Animal and Wildlife Clinic, Faculty of Veterinary Medicine, Chiang Mai University, 155 Mae Hia, Mueang, Chiang Mai 50100 Thailand; 2 Research Center of Producing and Development of Products and Innovations for Animal Health and Production, Chiang Mai University, Chiang Mai, 50100 Thailand; 3 Faculty of Veterinary Medicine, Chiang Mai University, 155 Mae Hia, Mueang, Chiang Mai 50100 Thailand

**Keywords:** Infectious-disease epidemiology, Risk factors, Urological manifestations

## Abstract

Feline lower urinary tract disease (FLUTD) is a common problem in cats. The objectives of the study were to determine the prevalence, clinical signs, and causes of FLUTD and the risk factors for FLUTD. The medical records of 3486 cats visiting Chiang Mai University Small Animal Veterinary Teaching Hospital (VTH) between November 2016 and October 2017 were reviewed. An age-matched case-control study was performed to determine the risk factors for FLUTD by comparing 78 cats with FLUTD and 78 clinically normal age-matched cats. For each animal, potential risk data were obtained from medical records and cat owner interviews; these were analysed for associations with FLUTD. Multivariable logistic regression analysis was performed to estimate the odds ratios and to adjust for expected confounding factors. The prevalence of FLUTD in cats visiting the Chiang Mai University Veterinary Teaching Hospital was 2.2%. The most common clinical signs identified were urethral obstruction (55.1%) and haematuria (23.1%). The most common diagnoses were feline idiopathic cystitis (FIC) (57.7%) and urolithiasis (struvite) (18%). The multivariable logistic regression analysis results indicated that FLUTD was most likely to be diagnosed in castrated male cats. FIC and urolithiasis were the most common diagnoses in cats with FLUTD, and male sex and castration increased the risk of FLUTD.

## Introduction

Feline lower urinary tract disease (FLUTD), a common disease in cats, is a syndrome that affects the reproductive tract, urinary bladder or urethra. The clinical signs of FLUTD are dysuria, haematuria, stranguria, pollakiuria and periuria. The prevalence of FLUTD among cats that had visited animal hospitals and clinics was 1.5% in the United States^1^ and 2.2% in Bangkok, Thailand^2^. FLUTD can be obstructive or non-obstructive. Obstructions may occur as inflammatory debris from feline idiopathic cystitis (FIC), urethral plugs or uroliths. Non-obstructive causes include FIC, uroliths, urothelial carcinoma, anatomic defects (such as urethral stricture) and urinary tract infection (UTI)^3^.

The risk factors for FLUTD differ across countries due to geography, season, diets^4^ and cats’ lifestyle. In New Zealand, low activity and indoor lifestyles were identified as risk factors for FLUTD^5^. In Norway, FLUTD was commonly found in male cats with an indoor lifestyle^6^. In Belgium, indoor confinement was the common risk factor for FLUTD^7^. In Bangkok, Thailand, a diet of commercial dry food and overweight were found to increase the risk for FLUTD^2^. In Austria, overweight cats had an increased risk for FLUTD, but the type of diet was not associated with FLUTD^8^.

The Small Animal Veterinary Teaching Hospital in Chiang Mai, Thailand, receives a substantial number of feline patients each year. No one has yet evaluated the risk factors associated with FLUTD in this region. Therefore, the objectives of the present study were to determine the prevalence, clinical signs, and causes of FLUTD and risk factors for FLUTD in cats in Chiang Mai, Thailand.

## Materials and Methods

### Case and control selection

Medical records of feline patients visiting the Small Animal Veterinary Teaching Hospital, Faculty of Veterinary Medicine, Chiang Mai University from November 2016 to October 2017 were reviewed. Cats were diagnosed with FLUTD and considered “cases” if they presented with dysuria, haematuria, stranguria, pollakiuria or periuria. The causes of FLUTD were identified by radiography, ultrasound, urinalysis or urine culture. Cats were considered clinically normal if the results of a physical exam by the veterinarian on duty and blood tests were both normal. These cats were aged-matched to the cases and used as controls. The age-matched controls were cats that visited the hospital for spaying, neutering or vaccinations during the period of case selection. The clinically normal cats were excluded if they had previously been diagnosed with FLUTD or other diseases. All cats were included without regard to sex or breed.

The protocol was approved by the Faculty of Veterinary Medicine, Chiang Mai University Animal Care and Use Committee (FVM-ACUC) (approval number: S17/2560).

### Data collection

Demographic data and risk factors were collected through questionnaires using face-to-face or telephone interviews with the cats’ owners. Data collected included age, sex, breed, types of food, water sources, frequency of feeding and drinking, number of litter boxes, number of cats in a household and lifestyle. The types of food included canned food, dry food and homemade food. The water sources were filtered water, underground water, bottled water and tap water. The lifestyles were indoor housing, outdoor housing or access to the outdoors and outdoor hunting.

### Statistical analysis

Overall 95% confidence intervals (95% CIs) were calculated. Age and body weight data are presented as the means ± standard deviations (SDs). The differences in age and body weight between groups were compared using Student’s *t-*test or the Mann-Whitney U test. Relative frequencies were used to describe the breed, sex and age of cats that developed FLUTD. Associations of risk factors with FLUTD were analysed using the Pearson chi-square test or Fisher’s exact test as appropriate. Odds ratios (ORs) and 95% CIs were estimated to determine the strength of association using univariate logistic regression. A multivariate logistic regression model was constructed using a backward stepwise elimination procedure against FLUTD in cats. Variables associated with FLUTD at *P*-values of ≤0.1 were included in the multivariate logistic regression analysis. Variables were retained in the model based on the likelihood ratio χ^2^ statistic at *P*-values of <0.05. All statistical analyses were performed using Epi Info version 7.1.5.0.

### Ethical approval

All applicable international, national and institutional guidelines for the care and use of animals were followed. All procedures performed in studies involving animals were in accordance with the ethical standards of the institution at which the studies were conducted.

## Results

Between November 2016 and October 2017, 3486 cats visited Chiang Mai University Small Animal Veterinary Teaching Hospital, and 78 (2.24%) of these were diagnosed with FLUTD.

**Figure 1:**
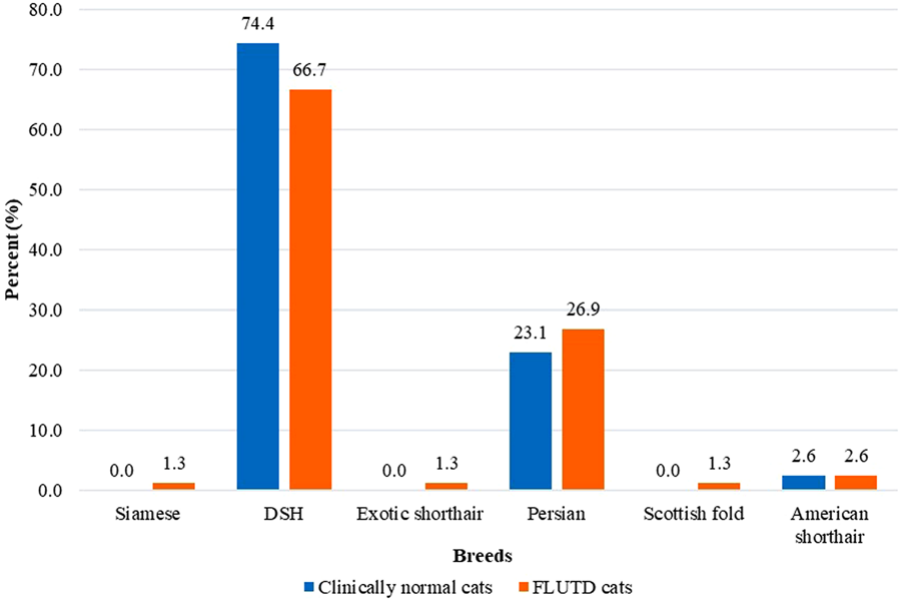
Proportion of clinically normal age-matched cats (n = 78) and cats with feline lower urinary tract disease (n = 78) by breed. DSH, domestic shorthair.

**Figure 2:**
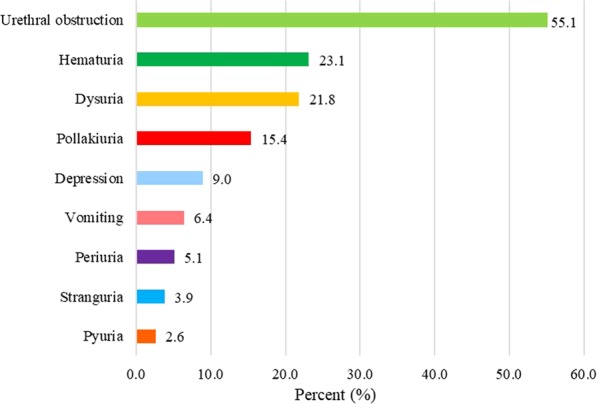
Clinical signs of feline lower urinary tract disease (n = 78) in cats seen at the Small Animal Veterinary Teaching Hospital, Chiang Mai University, between November 2016 and October 2017.

**Figure 3:**
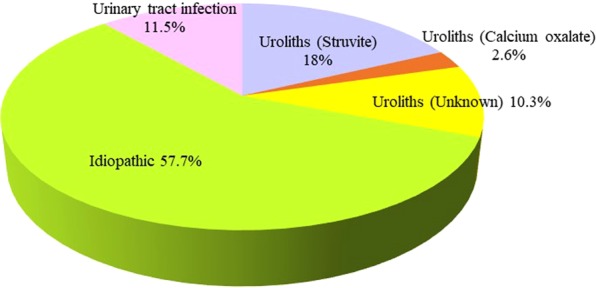
Causes of feline lower urinary tract disease (n = 78) in cats seen at the Small Animal Veterinary Teaching Hospital, Chiang Mai University, between November 2016 and October 2017.

The study population included 78 cats with FLUTD and 78 normal (control) cats. FLUTD was commonly found in cats of the domestic shorthair (52, 66.7%), Persian (21, 26.9%), American shorthair (2, 2.6%), exotic shorthair (1, 1.3%), Scottish fold (1, 1.3%) and Siamese (1, 1.3%) breeds (Fig. 1). The clinical signs noted upon presentation were urethral obstruction (43, 55.1%), haematuria (18, 23.1%), dysuria (17, 21.8%), pollakiuria (12, 15.4%), depression (7, 9.0%), vomiting (5, 6.4%), periuria (4, 5.1%), stranguria (3, 3.9%) and pyuria (2, 2.6%) (Fig. 2). The most frequent diagnosis was FIC (45, 57.7%), followed by struvite urolithiasis (14, 18.0%), urinary tract infection (UTI) (9, 11.5%), unknown urolithiasis (8, 10.3%) and calcium oxalate urolithiasis (2, 2.6%) (Fig. 3; Table 1).

**Table 1:** Causes of feline lower urinary tract disease (n = 78) in cats seen at the Small Animal Veterinary Teaching Hospital, Chiang Mai University, between November 2016 and October 2017.

	Idiopathic (n = 45)	Urinary tract infection (n = 9)	Uroliths (Struvite) (n = 14)	Uroliths (Calcium oxalate) (n = 2)	Uroliths (Unknown) (n = 8)
**Age**					
1–2 years	18	5	8	0	1
3–4 years	13	3	5	2	2
5–6 years	10	0	1	0	2
>7 years	4	1	0	0	3
**Sex**					
Female intact	1	0	0	0	0
Female spayed	3	1	0	0	3
Male intact	18	6	6	2	2
Male castrated	23	2	8	0	3
**Breeds**					
Domestic shorthair	30	7	9	1	5
Siamese	0	0	0	1	0
Persian	11	2	5	0	3
Scottish fold	1	0	0	0	0
Exotic shorthair	1	0	0	0	0
American shorthair	2	0	0	0	0

These cats included 34 intact males (43.6%), 36 castrated males (46.2%), 1 intact female (1.3%) and 7 spayed females (9.0%). The mean age and body weight of the cats with FLUTD (3.95 ± 2.33 years old and 4.44 ± 1.07 kilograms (kg), respectively) were not significantly different from those of the clinically normal age-matched cats (3.86 ± 2.57 years old and 4.13 ± 1.17 kg, respectively) (Table 2). Using the chi-square or Fisher’s exact test, sex, the drinking water source and number of litter boxes were found to be significantly associated with FLUTD (Table 3).

**Table 2:** Mean age and body weight between clinically normal age-matched cats and cats with FLUTD.

Parameter	Units	Clinically normal cats (n = 78)	Cats with FLUTD (n = 78)	*P*-value
Age	years	3.86 ± 2.57	3.95 ± 2.33	0.83
Body weight	kg	4.13 ± 1.17	4.44 ± 1.07	0.08

FLUTD, Feline lower urinary tract disease.

**Table 3:** Univariate logistic regression analysis of variables associated with feline lower urinary tract disease in case (n = 78) and control (n = 78) cats that visited the Chiang Mai University Small Animal Veterinary Teaching Hospital between November 2016 and October 2017.

Variables	Clinically normal cats (n = 78)	Cats with FLUTD (n = 78)	Odds ratio	95% CI	*P*-value
**Sex**^a^					**0.003**
Female intact	12/78 (15.4%)	1/78 (1.3%)	Reference		
Female spayed	13/78 (16.7%)	7/78 (9.0%)	6.46	0.69–60.53	0.102
Male intact^a^	30/78 (38.5%)	34/78 (43.6%)	13.60	1.67–110.85	**0.015**
Male castrated^a^	23/78 (29.5%)	36/78 (46.2%)	18.78	2.29–154.28	**0.006**
**Breeds**					0.615
Domestic shorthair	58/78 (74.4%)	52/78 (66.7%)	Reference		
Siamese	0/78 (0.0%)	1/78 (1.3%)	N/A		
Persian	18/78 (23.1%)	21/78 (26.9%)	1.24	0.59–2.62	0.570
Scottish fold	0/78 (0.0%)	1/78 (1.3%)	N/A		
Exotic shorthair	0/78 (0.0%)	1/78 (1.3%)	N/A		
American shorthair	2/78 (2.6%)	2/78 (2.6%)	0.00	0.00–undefined	0.970
**Type of diets**					0.054
Dry and canned food	37/78 (47.4%)	21/78 (26.9%)	Reference		
Homemade food	0/78 (0.0%)	2/78 (2.6%)	N/A		
Homemade & dry food	8/78 (10.3%)	10/78 (12.8%)	2.20	0.75–6.44	0.149
Dry food^a^	26/78 (33.3%)	39/78 (50.0%)	2.64	1.27–5.48	**0.009**
Dry, canned, and homemade food	7/78 (9.0%)	6/78 (7.7%)	1.51	0.45–5.09	0.506
**Frequency of meals**^**a**^					0.162
1	0/78 (0.0%)	1/78 (1.3%)	N/A		
2	42/78 (53.9%)	31/78 (39.7%)	Reference		
3	3/78 (3.9%)	5/78 (6.4%)	2.26	0.50–10.17	0.289
4	2/78 (2.6%)	0/78 (0.0%)	N/A		
Ad libitum	31/78 (39.7%)	41/78 (52.6%)	1.79	0.93–3.46	0.082
**Drinking water source**					0.051
Filtered water	22/78 (28.2%)	7/78 (9.0%)	Reference		
Underground water	0/78 (0.0%)	3/78 (3.9%)	N/A		
Bottled water	38/78 (48.7%)	33/78 (42.3%)	2.50	0.92–6.85	0.074
Tap water^a^	18/78 (23.1%)	35/78 (44.9%)	2.93	1.07–8.04	**0.037**
**Frequency of drinking water**					≅1.000
Ad libitum	76/78 (97.4%)	76/78 (97.4%)	Reference		
2	2/78 (2.6%)	2/78 (2.6%)	1.00	0.14–7.28	≅1.000
**Life style**					0.544
Indoor and outdoor	10/78 (12.8%)	6/78 (29.5%)	Reference		
Outdoor	20/78 (25.6%)	23/78 (29.5%)	1.92	0.59–6.21	0.280
Indoor	48/78 (61.5%)	49/78 (62.8%)	1.70	0.57–5.05	0.338
**Number of cats in the household**					0.511
1 cat	22/78 (28.2%)	17/78 (21.8%)	Reference		
2 cats	17/78 (21.8%)	15/78 (19.3%)	1.41	0.45–2.92	0.782
≥3 cats	39/78 (50.0%)	46/78 (59.0%)	1.53	0.71–3.27	0.278
**Number of litter boxes**^**a**^					**0.011**
≥no. of cats	39/78 (50.0%)	18/78 (23.1%)	Reference		
<no. of cats^a^	39/78 (50.0%)	60/78 (76.9%)	2.44	1.22–4.88	**0.011**

FLUTD, Feline lower urinary tract disease; 95% CI, 95% confidence interval; N/A, not available.

^a^*p* < *0.05*.

The univariable logistic regression results (Table 3) indicate that intact male and castrated male cats had a higher risk for FLUTD than intact females (OR = 13.60, 95% CI: 1.67–110.85; OR = 18.78, 95% CI: 2.29–154.28, respectively). Cats fed dry food had a 2.64 times higher risk for FLUTD than those fed both dry and canned food (OR = 2.64, 95% CI: 1.27–5.48). Compared with drinking filtered water, drinking tap water was associated with an increased risk for FLUTD (OR = 2.93, 95% CI: 1.07–8.04). Compared with having at least as many litter boxes as cats in the household, having fewer litter boxes than cats in the household was associated with an increased odds ratio for FLUTD (OR = 2.44, 95% CI: 1.22–4.88). The multivariate logistic regression analysis in the present study demonstrated that intact male status, castrated male status, tap water drinking and the presence of fewer litter boxes than cats were associated with a higher risk for FLUTD than were the other factors (OR = 3.11, 95% CI = 1.16–8.33; OR = 4.45, 95% CI = 1.58–12.48; OR = 3.78, 95% CI = 1.31–10.89; OR = 3.28, 95% CI = 1.55–6.91, respectively) (Table 4).

**Table 4:** Multivariate logistic regression analysis (backward) of variables including intact male status, castrated male status, a dry food diets, ad libitum feeding, bottled water drinking, tap water drinking and the presence of fewer litter boxes than cats in age-matched cats with FLUTD at Chiang Mai University Small Animal Veterinary Teaching Hospital between November 2016 and October 2017.

Variables	Odds ratio	95% CI	*P-value*
Male intact^*^	3.11	1.16–8.33	**0.023**
Male castrated^*^	4.45	1.58–12.48	**0.005**
Tap water^*^	3.78	1.31–10.89	**0.014**
Number of litter boxes <number of cats^*^	3.28	1.55–6.91	**0.002**

FLUTD, Feline lower urinary tract disease; 95% CI, 95% confidence interval.

^*^p < *0.05*.

## Discussion

The prevalence of FLUTD in this study (2.24%) was similar to that in previous studies in the United States (1.5%)^1^ and Bangkok, Thailand (2.2%)^2^, but much lower than the 8% reported for the United States and Canada from 1980 to 1997^4^. The discrepant findings may be due to differences in the geographic area, diet, popular breeds of cats in each country and duration of sample collection.

The ages of the cats with FLUTD (range, 1.02 to 12.03 years old) were matched with those of the clinically normal cats (range, 1.20 to 11.60 years old). In this study, FLUTD was diagnosed in cats aged 1 to 6 years old. This result was similar to that identified in previous research^4,9^. Willeberg and Priester (1976) reported that cats affected with FLUTD were 2 to 6 years old^9^. The most common causes of FLUTD in cats aged 2 to 7 years old were urethral plugs and anatomical defects^4^. This study included a larger proportion of young cats than did other studies^4,9^. Most cats with FLUTD in this study were 1 to 2 years old (41.0%). These cats were diagnosed with FIC and UTI. While a previous study in Thailand demonstrated that FLUTD has also been diagnosed in cats older than 10 years^2^, the causes in these cats were more commonly UTI, stones and neoplasia. However, we found no significant differences between the cats with FLUTD and clinically normal cats in each age range. It is possible that there is a wide disparity in the prevalence among studies due to the populations evaluated, historical period and diet.

FLUTD has also been reported in Germany, where the most common diagnosis was FIC (55.0%), followed by bacterial UTI (18.9%)^10^. Older cats are often afflicted with systemic diseases, such as chronic kidney disease (CKD), diabetic mellitus (DM) or hyperthyroidism. Several studies have reported that UTIs are common in cats with CKD^11^ and diabetes^12^. UTIs also affect older cats^13^. However, this study found that young cats were more likely to develop UTIs. Young cats with UTI often have other pre-existing complications, such as kidney disease. Two of the young cats with UTIs in this study had polycystic kidneys. This condition may have contributed to the higher prevalence of UTIs in young cats than in older cats. These results suggest that veterinarians need to identify the other concurrent problems in young cats with UTIs.

Several previous studies found that overweight cats had an increased risk of FLUTD^4,7,9,14–16^. Pusoonthornthum *et al*. (2012) reported that the risk of FLUTD was 4 times higher in overweight than in normalweight cats^2^. One may speculate that overweight cats are less active, possibly void less frequently and drink less water. Moreover, obesity may cause fat accumulation around the urethra and penis, resulting in urethral compression and increased urinary dysfunction. Other studies have shown that cats with FIC are more likely to be ‘stress eaters’ and move little because they are stressed, further exacerbating their obesity. However, the mean body weight of the cats with FLUTD in this study was not significantly higher than that of the clinically normal cats. The inclusion criteria designated for the age-matched case-control analysis in this study may have resulted in the difference in the mean body weight between the present study and the previous study. In the previous study, the immature cats less than 1 year old in the clinically normal group (31.4%) affected the mean body weight in that group, implying that overweight cats are likely to have an increased risk for FLUTD^2^. Cats less than 1 year old were not included in this study.

Willeberg and Priester (1976) reported that neutering was associated with an increased risk for FLUTD in both male and female cats^9^. This study found that most cats with FLUTD were castrated males; this factor significantly increased the risk for FLUTD. Similarly, others have reported that male cats were at higher risk of FLUTD^6,17^ and FIC^14,18^ than female cats. Non-spayed female cats exhibited a decreased risk of FLUTD^4^. Cats with FLUTD are more often male or neutered^6,9^ than female or intact due to the narrowness and curvature of the penile urethra. Castration affects the density of the elastic and collagen fibres in the periurethral tissues; this decreases the compliance of the periurethral region^19^. Moreover, most castrated male cats were less active, leading to weight gain, a common risk factor for FLUTD. This may explain why FLUTD was more prevalent in males than females. Neutering cats offers several advantages, including a reduction in territorial behaviours (fighting and marking)^20^ and population control. If castration increases the risk for FLUTD, as indicated in this study, then attention must be devoted to reducing the other risks for the development of this complex in intact cats.

In this study, the breed of cat was not associated with FLUTD. Several previous studies also found no association between cat breed and FLUTD^2,6,7,21^. However, others reported a positive association between Persian or other long-haired cat breeds and FLUTD^5,9,14^. In the United States and Canada, purebred cats, including Russian Blues, Himalayans, Persians, Abyssinians and Manxes, had an increased risk of FLUTD^4^. In Poland, non-pedigree cats were at risk for FLUTD^17^. The variability in the results is probably due to the popularity of particular cat breeds at different times. This variability probably shows that in areas where pedigree cats are more common, they become affected.

A dry food diet was associated with only a 2.64 times greater risk for FLUTD than a diet including both dry and canned food (OR = 2.64, 95% CI: 1.27–5.48). This finding agreed with previous studies that found that dry food was associated with an increased risk for FLUTD^2,5,9^. However, one study reported that there was no significant difference in the diet between cats with FLUTD and clinically normal cats^17^. Cats eating only dry food consume less water than cats eating canned food because most cats consume water directly through their food^22^. Drinking insufficient amounts of water can lead to FLUTD by increasing the chances of crystal formation from concentrated urine. Similarly, cats with FLUTD that ate only dry food were more likely to have urolithiasis (13/39, 33.3%).

In this study, most cats with FLUTD presented with urethral obstruction, as has been reported in some^10,17^ but not all previous studies. Other clinical signs varied by study and depended on the lifestyle of the cats, including indoor or outdoor husbandry. An owner’s ability to observe clinical signs is affected by many factors, including outdoor access, the number of cats and the owner’s work schedule. Furthermore, only the prominent clinical signs of FLUTD, such as dysuria or haematuria, can be easily detected in outdoor cats.

Feline idiopathic cystitis (FIC) was the most common diagnosis in this study, followed by urolithiasis and UTI. Our results were similar to several studies that showed cats affected by FLUTD were most often diagnosed with FIC, urolithiasis and UTI^17,23^. The factors that contributed to the concordance of the results between the present study and the previous studies were male sex, a dry food diet^17,23^ and a strictly indoor lifestyle^23^.

Our study demonstrated that cats that drank tap water had a significantly higher risk of FLUTD than those that drank filtered water. However, a previous study outside Thailand did not find an association between FLUTD and the water source^2^. In contrast to another study^2^, the present study did not show an association between the frequency of drinking and the risk of FLUTD. Most clinically normal age-matched and cats with FLUTD in this study had ad libitum access to drinking water (97.4% and 97.4%, respectively).

Previous studies found that overpopulation was one of the most common causes of FLUTD in cats^5,14,18^. Given the solitary nature of cats, overcrowding may result in abnormal behaviour. Cats with FIC, which is commonly diagnosed in cats with FLUTD, are reported to have a neuroendocrine imbalance^24–27^ due to a mild decrease in the size of the adrenal gland^28^ that makes them more sensitive to stressful situations^29^. Modifying a multimodal environment or decreasing the stress in an environment are also reported as adjunctive therapies for cats with FIC^21^. However, in this study, the number of cats in the household was not associated with FLUTD.

Inadequate numbers of litter boxes for the number of cats in a household increased the risk of FLUTD^30^. FLUTD is related to litter box management problems, including inappropriate litter box usage^5,7^ and litter boxes that are too small^14^. Good management should include appropriate litter box cleanliness^31^, a sufficient number of litter boxes^26^, and properly sized, shaped and located litter boxes. Inappropriate litter box management, such as: not regularly cleaning the litter box, could cause FLUTD in some cats, which then leads to abnormal voiding behaviour, such as infrequent urination^32^. Normal urination patterns of cats are very important. Therefore, providing an appropriate number of litter boxes, i.e., more litter boxes than cats, may also reduce inappropriate elimination behaviour that can lead to FLUTD. However, very few studies have researched litter box management for cats. Sharing the same litter box can lead to inappropriate elimination behaviour because the urine and faeces in the litter box are from an unfamiliar individual^33^. One study reported the relationship between urine and faecal odour and litter box preferences^34^. However, another study demonstrated that cats were not averse to sharing a litter box^35^. The maintenance of litter boxes is the most important factor, especially in multicat households.

In conclusion, FIC was one of the most commonly diagnosed urologic problems in a retrospective study of cats seen at the teaching hospital at the Faculty of Veterinary Medicine, Chiang Mai University, Thailand. Young cats with UTIs were commonly affected by complex problems, such as polycystic kidneys. Intact male status, castrated male status, a dry food diet, tap water as a drinking water source and the presence of fewer litter boxes than cats were risk factors for FLUTD. Future studies are needed to better identify and manage the causes of FLUTD.
